# Tumor-infiltrating immune cell status predicts successful response to immune checkpoint inhibitors in renal cell carcinoma

**DOI:** 10.1038/s41598-022-24437-6

**Published:** 2022-11-27

**Authors:** Akira Kazama, Vladimir Bilim, Masayuki Tasaki, Tsutomu Anraku, Hiroo Kuroki, Yuko Shirono, Masaki Murata, Kaede Hiruma, Yoshihiko Tomita

**Affiliations:** 1 Department of Urology, Division of Molecular Oncology, Niigata University Graduate School of Medical and Dental Sciences, Niigata, 951‑8510 Japan; 2 Kameda Daiichi Hospital, Niigata, 950-0165 Japan

**Keywords:** Cancer, Immunology, Oncology, Urology

## Abstract

Immune checkpoint inhibitors (ICI) have dramatically changed the treatment of metastatic renal cell carcinoma (mRCC). Although many studies have reported biomarkers as predicting the efficacy of ICI in mRCC, they remain controversial and have challenges to apply in real-world practice. We evaluated prognostic significance of multiple molecules associated with tumor immunity in patients treated with ICI. The molecules were detected in tumor tissues by immunohistochemical staining. We identified CD8-positive T cells and CD68-positive macrophages infiltrating into the tumor tissue as significant favorable prognostic factors for ICI treatment. Conversely, high expression of CD4-positive T cells was associated with poor response to ICI. Furthermore, we demonstrated that scoring for the expression status of these three molecules provides a remarkably accurate biomarker in patients with mRCC. Even the classical approach of immunohistochemistry could predict the outcome of ICI treatment by assessing the combined status of tumor-infiltrating immune cells.

## Introduction

Renal cell carcinoma (RCC) accounts for 3–5% of all carcinomas. According to the World Health Organization, approximately 180,000 individuals succumb to the disease annually^1^. Systemic treatment is required for 25% of RCC patients with metastases detected at the time of initial diagnosis and for > 10% of RCC patients experiencing recurrence following the surgical resection of the tumor^2^. The treatment for metastatic renal cell carcinoma (mRCC) has made remarkable progress in recent years, and many clinical trials have indicated that immune checkpoint inhibitor (ICI) is the mainstay of mRCC treatment. Several clinical trials have reported that first-line therapy with ICI combination or ICI and Tyrosine kinase inhibitor (TKI) has a 39–70% objective response rate^3–7^, and second-line or further treatment has a 25%^8^. Identifying biomarkers predicting treatment response in ICI is a significant issue for appropriate drug selection and reduction of unnecessary adverse events.

Previously, several studies reported on potential biomarkers of ICI treatment for RCC, including high PD-L1 expression^9^, high tumor mutation burden (TMB)^10^, CD8-positive T-cell infiltration, genetic mutations such as PBRM1^11^ and BRAF^12^, and the tumor microenvironment^13^. PD-L1 expression and high TMB are useful biomarkers in other solid tumors, but there are controversial reports in RCC^14,15^. In addition, with the advancement of next-generation sequencing and mass cytometry-based clustering, studies from gene expression analysis, and T cell signature analysis by single-cell sequence have been reported as useful tools to determine biomarkers^16^. However, there is still the time and cost disability to performing these analyses in individual mRCC patients in real-world clinical settings. Therefore, we used a relatively simple immunohistochemical approach to analyze immune-related molecules expressed in tumor tissues and tumor-infiltrating immune cells to identify prognostic biomarkers for ICI treatment.

## Materials and methods

### Patients and specimens

To study immune-related molecules expressed in RCC tissues and the therapeutic effects of ICI, 72 patients who received ICI treatment for metastatic or advanced RCC at Niigata University Medical and Dental Hospital between January 2015 and December 2020 were included in the study. Of these, 60 patients for whom clinical pathology data and tissue specimens were available were reviewed. This study was done following the guidelines from the Helsinki Declaration and approved by the Ethics Committee of Niigata University (approval number: 2018-0348). Written informed consent for the study was obtained from all patients or their families. All patients with RCC in this series underwent nephrectomy, partial nephrectomy, or percutaneous renal tumor biopsy. Patients were then treated with systemic therapy, including ICI, for recurrence or metastasis of RCC and were followed up with periodic imaging evaluation to determine response to therapy. A total of 60 RCC samples were collected by resection or biopsy of the primary tumor and used for immunohistochemical staining (IHC) analysis. Clinical and pathological data, including age, gender, TNM stage, and World Health Organization / International Society of Urologic Pathologists (WHO/ISUP) grade, were obtained from hospital medical records. Progression-free survival (PFS) and overall survival (OS) were defined as the time from the start of ICI treatment to imaging or clinical progression and death, respectively. The median follow-up period was 16 months (range 1–71 months).

### Immunohistochemistry

**Figure 1:**
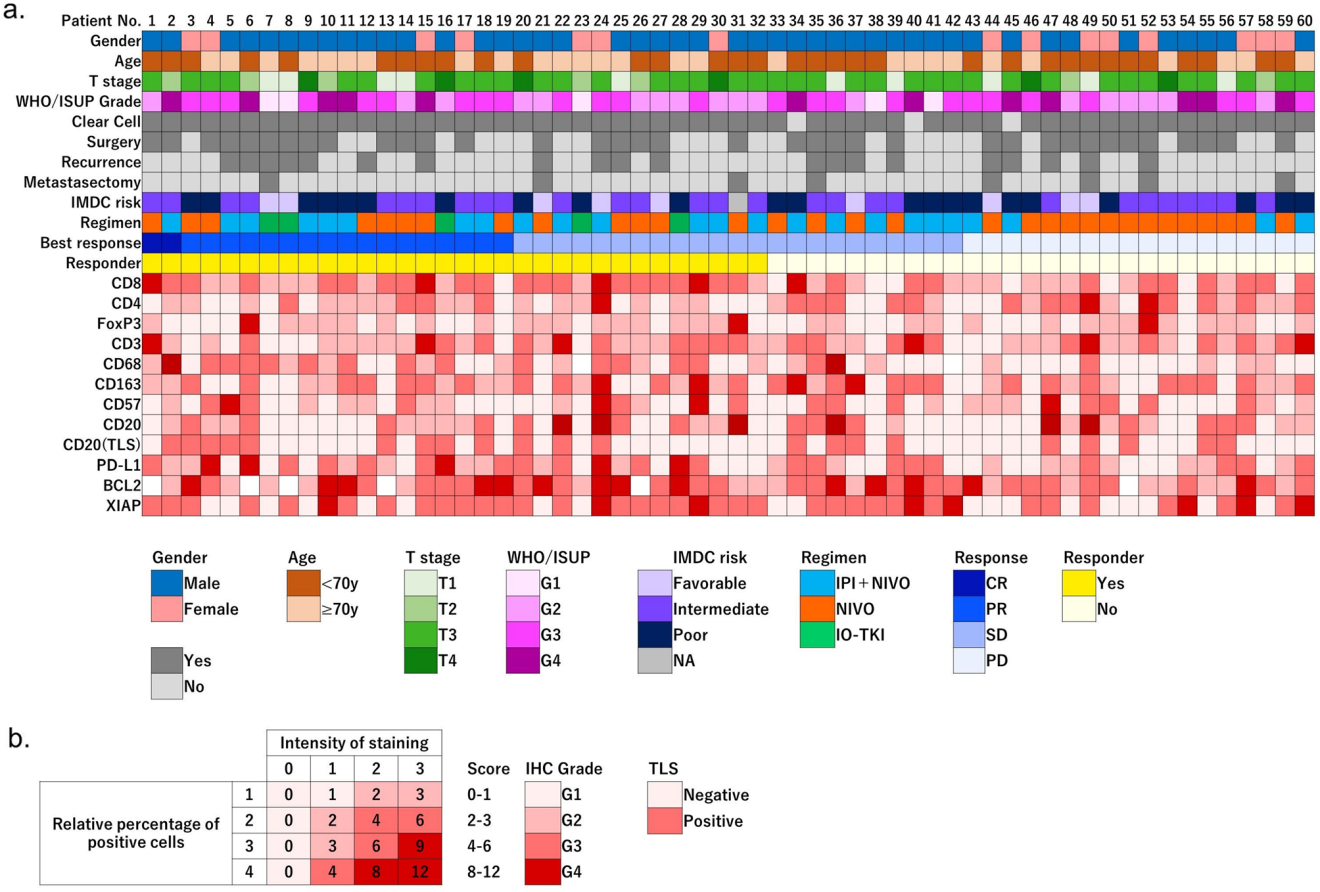
(**a**) The figure shows the background, clinicopathological characteristics, and IHC scores for all patient cohorts. Each item is color-coded and examples are given below the figure. (**b**) The calculation of the IHC score is shown. It was scored from 0 to 12 according to the percentage of positive cells and the intensity of staining. Staining grades were classified from 1 to 4 according to the total score. For tertiary lymphoid structures, status was graded as positive or negative.

Immunohistochemical staining was performed using formalin-fixed and paraffin-embedded RCC tissue specimens. In cases of nephrectomy, the center of the tumor, excluding necrotic tissue, was selected for specimen preparation. For biopsy cases, the entire obtained sample was prepared as a specimen. Slides were cut at a thickness of 4 μm. Paraffin sections were degreased with Xylene and rehydrated in a gradient series of ethanol solutions. Endogenous peroxidase was blocked with 3% hydrogen peroxide for 10 min. Subsequently, antigen activation was performed by autoclaving (121 °C, 20 min) using citrate buffer. The slides were then incubated with various primary antibodies (Supplementary S1) for 1 h at 25 °C and treated with Histofine simple stain MAX-PO (Nichirei, Tokyo, Japan) for 30 min at room temperature. The staining reaction was developed by DAB and nuclear counterstaining was performed with Mayer-Hematoxylin Solution (Fujifilm WAKO, Tokyo, Japan). Positive and negative controls were included in each staining series. The IHC results were evaluated by two independent urological pathologists who were experts and unaware of the patient's clinical condition. The calculation of the IHC score is shown in Fig. 1b. It was scored from 0 to 12 according to the percentage of positive cells and the intensity of staining. Staining grades were classified from 1 to 4 according to the total score; absent or very weak (Garade1), weak (Grade2), moderate (Grade3), and strong (Grade4) respectively. Immune cells were evaluated for infiltration into the tumor tissue, excluding immune cells in the stroma or at the tumor margins.

### Statistical analysis

All statistical analyses were performed using SPSS version 19.0 software and EZR software. The association between clinicopathological variables, response to ICI treatment, and IHC expression status of immune-related molecules and response to ICI treatment were each evaluated with Fisher’s exact test. Kaplan–Meier method with log-rank test was used to estimate prognosis after ICI treatment stratified by IHC expression status. Univariate analysis and multivariate analysis of the association with FPS and OS were performed by Cox proportional hazard model in each molecule, and the significance level was determined by a log-rank test. The following molecular biomarkers, CD8 + , CD4 + , and CD68 + , were found significant predictors of response to immune checkpoint inhibitors in this cohort of patients (Table 2). These factors were selected for multivariate analysis (Table 3). Survival outcomes were estimated as hazard ratio (HR) and their 95% confidence interval (CI); P < 0.05 was considered statistically significant.

## Results

The patient clinicopathological backgrounds and the grade of immunohistochemical staining of all patients are shown in Fig. 1. Forty-five patients were male, and the median age was 68 years (range 23–81 years). Thirty-six patients underwent surgery including radical and partial nephrectomy, and 43 patients had T3 or higher T stage. The majority of the patients (57/60) had clear cell carcinoma. The histological type in the remaining 3 patients was chromophobe RCC (2/60) and unclassified RCC (1/60). Thirty-seven patients had WHO/ISUP grade 3 or higher. Forty patients had unresectable tumors or had metastases at the time of diagnosis. in 5 patients (pembrolizumab plus axitinib in 3 and nivolumab plus cabozantinib in 2 patients). The risk criteria for IMDC were favorable in 9, intermediate in 28, and poor in 22 patients, respectively. The best response after ICI treatment was complete response (CR) in 2, partial response (PR) in 17, stable disease (SD) in 23, and progression disease (PD) in 18 patients. The IHC staining was graded in four levels, with light red indicating low expression (score 0–1: Grade 1, score 2–3: Grade 2) and dark red indicating high expression (score 4–6: Grade 3, score 8–12: Grade 4).

Table1 shows the results of Fisher's exact test for the clinicopathological characteristics and treatment efficacy of ICI (Responders vs non-Responders). Patients with CR, PR, or SD ≥ 10 months were defined as responders and patients with PD or SD < 10 months were defined as non-responders. There was no significant association with ICI treatment response among patient characteristics such as age, nephrectomy, T stage, WHO/ISUP grade, recurrent case, metastatic resection, and IMDC risk. Only patients treated with ICI as first-line therapy have a trend that showed a higher response (p = 0.077).

**Table 1:** Baseline characteristics and Fisher's exact test results.

Characteristics	Total	Responder	Non-responder	p value
**Sex**				
Male	45	25	20	0.567
Female	15	7	8	
**Age**				
< 70	33	16	17	0.446
≥ 70	27	16	11	
**Nephrectomy**				
Yes	36	19	17	0.563
No	24	13	11	
**T stage**				
T1-2	17	10	7	0.775
T3-4	43	22	21	
**WHO/ISUP grade**				
G1-2	23	14	9	0.430
G3-4	37	18	19	
**Recurrence**				
Yes	20	11	9	1.000
No	40	21	19	
**Metastasectomy**				
Yes	8	3	5	0.454
No	52	29	23	
**1st line treatment**				
Yes	29	19	10	0.077
No	31	13	18	
**IMDC risk**				
Favorable	9	5	4	0.731
Intermediate	28	16	12	
Poor	22	10	12	
NA	1	1	–	

*ISUP* international society of urological pathology, *IMDC* international metastatic renal cell carcinoma database consortium.

Responders: patients with CR, PR, or SD ≥ 10 months.

Non-responders: patients with PD or SD < 10 months.

**Figure 2:**
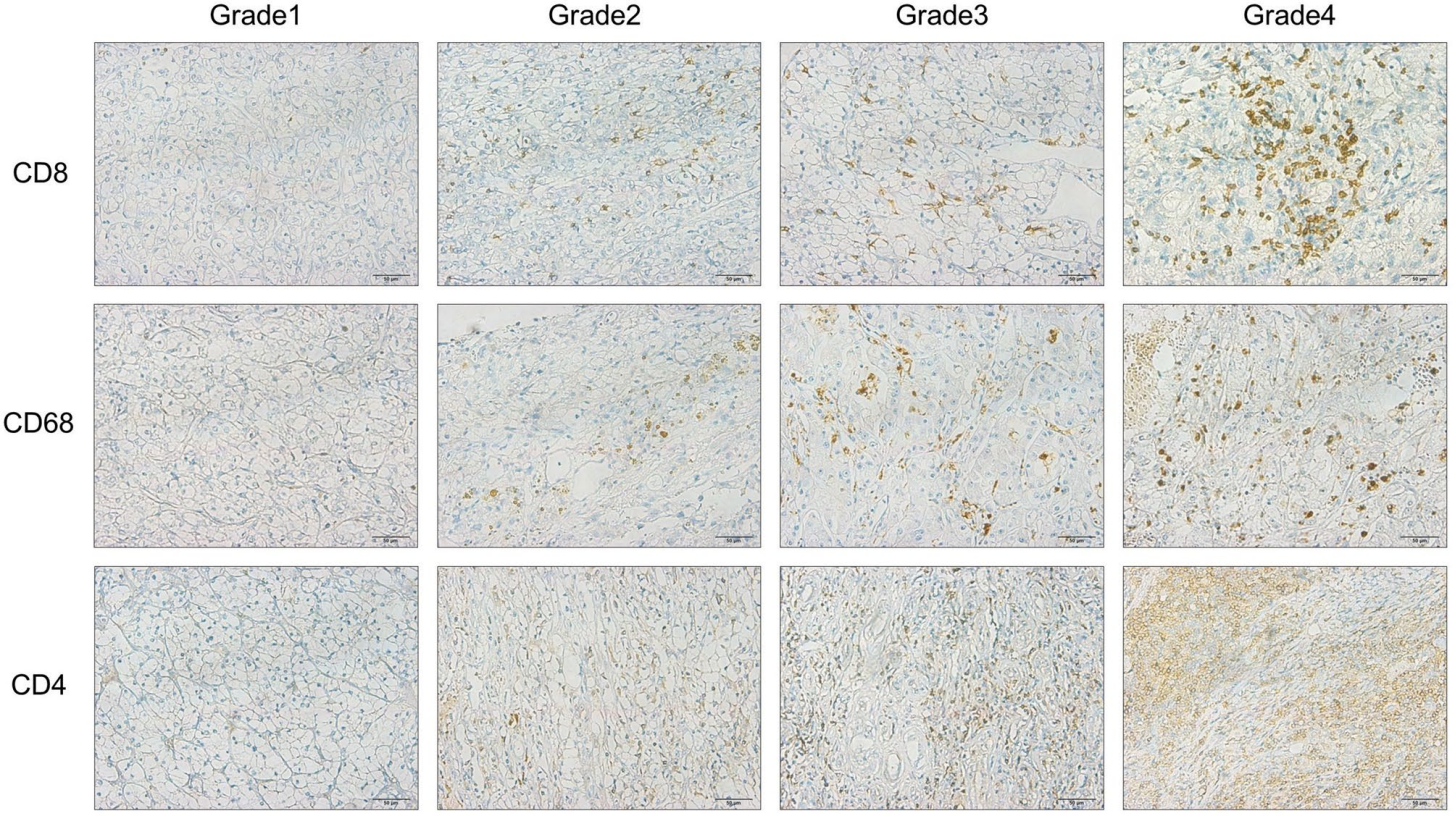
Representative immunohistochemical staining of CD8, CD68, and CD4 in tumor tissue obtained from RCC patients. All IHC Staining was defined as Grade1 when all tumor infiltrated cells were negative or no cancer cell was stained, Grade2 when staining was weakly positive, Grade3 when staining was moderately positive, and Grade4 when staining was strongly positive. Magnification is × 200 and × 400. Scale bar shows 100 μm and 50 µm, respectively.

Table 2 shows the relationship using Fisher's exact test between the IHC staining status (Grade 1–4) and the ICI treatment response. There was a significantly higher proportion of responders in the CD8 and CD68 high expression groups. (CD8; p = 0.037, CD68; p = 0.009). Representative images of immunohistochemical staining for CD8, CD68, and CD4 are shown in Fig. 2.

**Table 2:** Fisher's exact test results between immunohistochemistry status and response.

Molecular biomarker		Responder	Non-responder	p value
CD8	Positive	22	11	0.037
	Negative	10	17	
CD4	Positive	5	14	0.006
	Negative	27	14	
FoxP3	Positive	8	8	0.778
	Negative	24	20	
CD3	Positive	12	12	0.793
	Negative	20	16	
PD-L1	Positive	15	9	0.297
	Negative	17	19	
CD68	Positive	14	3	0.009
	Negative	18	25	
CD163	Positive	13	15	0.437
	Negative	19	13	
CD57	Positive	8	8	0.778
	Negative	24	20	
BCL-2	Positive	19	18	0.793
	Negative	13	10	
XIAP	Positive	15	12	0.799
	Negative	17	16	
CD20	Positive	10	10	0.787
	Negative	22	18	
TLS	Positive	12	9	0.788
	Negative	20	19	

*CD* cluster of differentiation, *FoxP3* forkhead box P3, *PD-L1* programmed death-ligand 1, *BCL2* B-cell/CLL lymphoma 2, *XIAP* X linked inhibitor of apoptosis protein, *TLS* tertiary lymphoid structures.

Responders: patients with CR, PR, or SD ≥ 10 months, non-responders: patients with PD or SD < 10 months.

**Figure 3:**
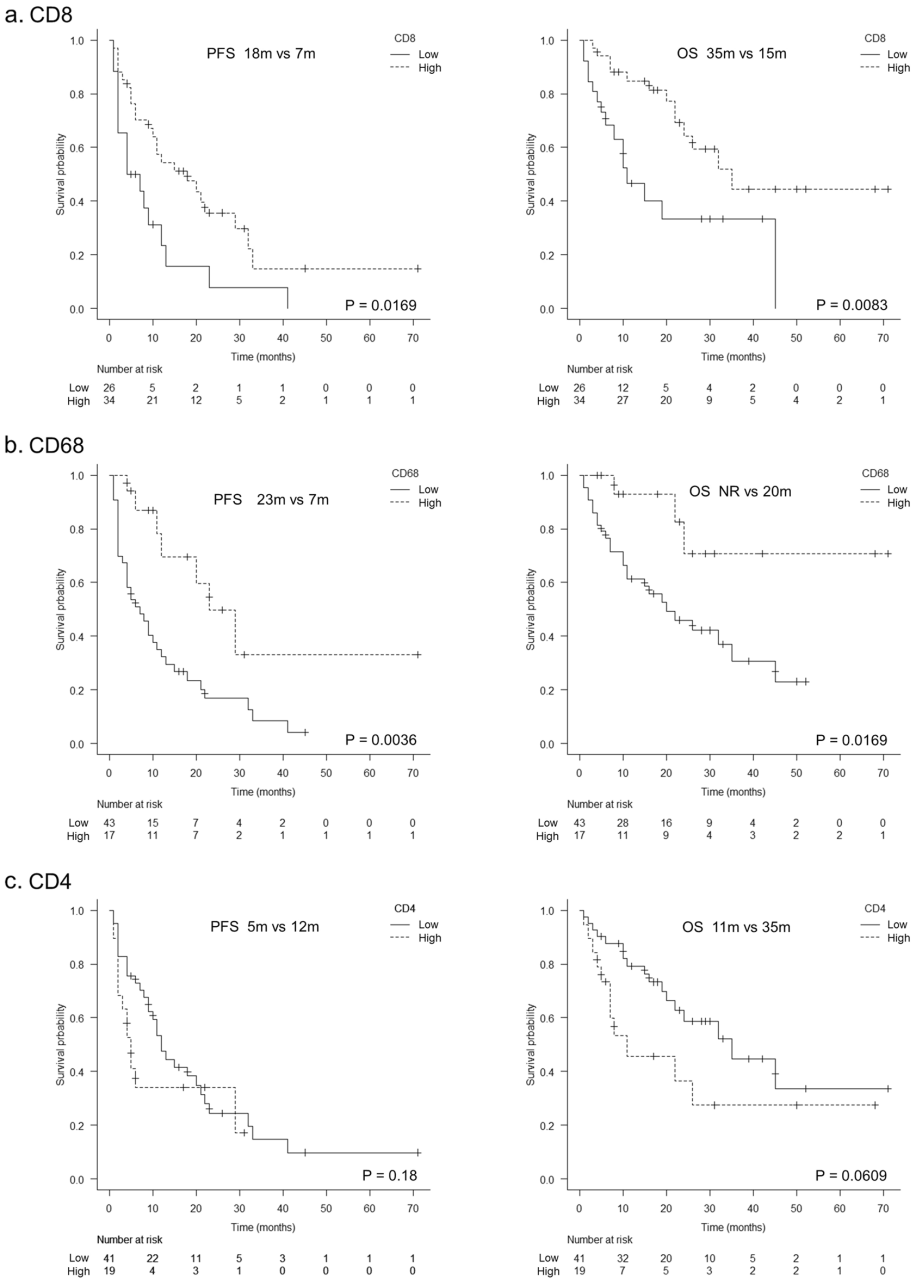
Kaplan–Meier plots of progression-free survival and overall survival stratified by IHC status of CD8, CD68, and CD4. (**a**) PFS and OS comparison by IHC staining of CD8 (log-rank P = 0.0169, P = 0.0083). (**b**) PFS and OS comparison stratified by IHC staining of CD68 (log-rank P = 0.0036, P = 0.0169). (**c**) PFS and OS comparison stratified by IHC staining of CD4 (log-rank P = 0.18, P = 0.0609).

**Figure 4:**
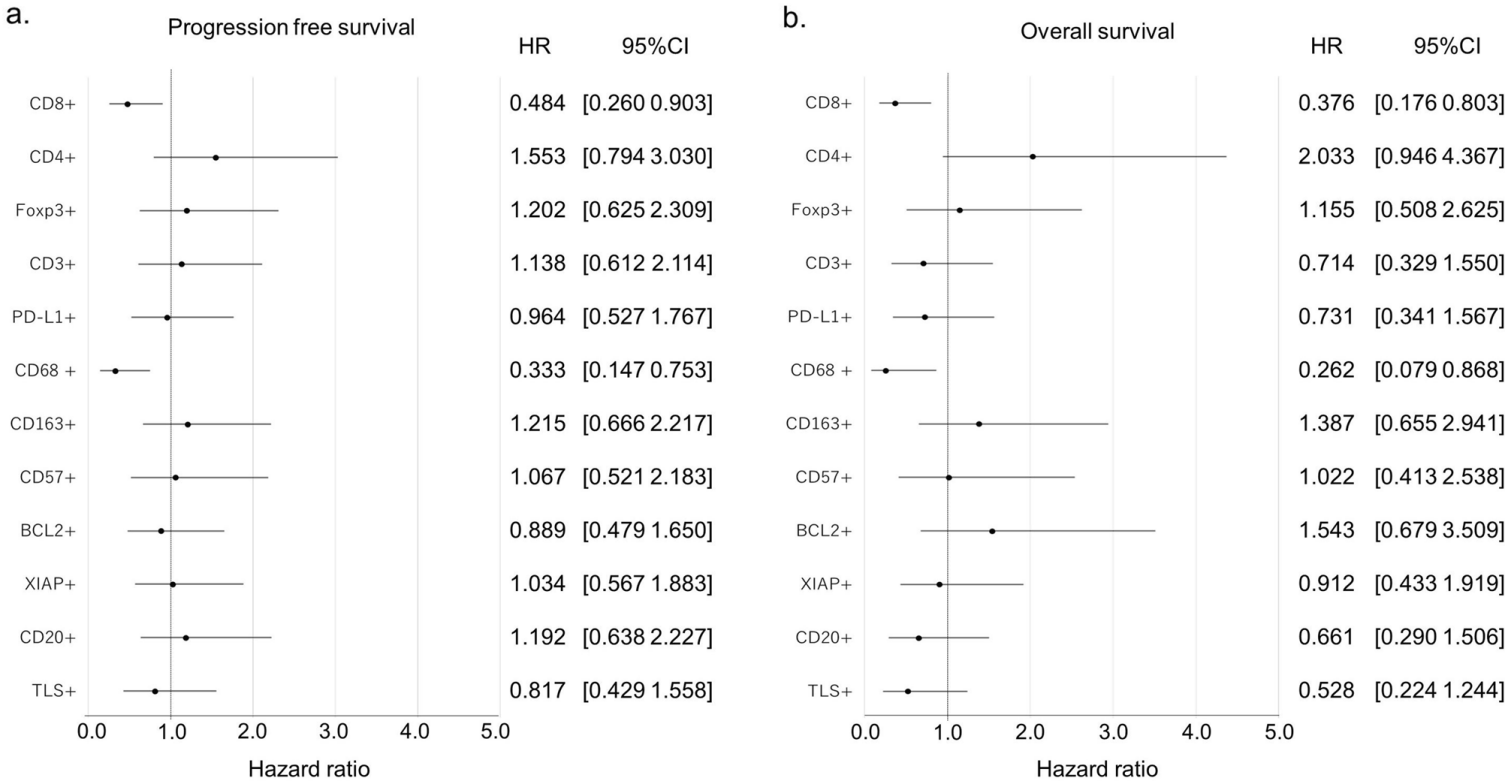
Forest plot diagram. (**a**) Hazard ratio (HR) with 95% confidence interval association between progression-free survival (PFS) and IHC staining status. (**b**) Hazard ratio (HR) with 95% confidence interval association between overall survival (OS) and IHC staining status.

According to the Kaplan–Meier survival analysis, high CD8 expression (18 vs 7 months; p = 0.0169) and high CD68 expression (23 vs 7 m; p = 0.0036) were significantly associated with long progression-free survival. Furthermore, for overall survival, high CD8 expression (35 m vs 15 m; p = 0.0083) and high CD68 expression (NR vs 20 m; p = 0.0169) were significantly associated with better survival. On the other hand, a trend toward shorter OS was observed in patients with high CD4 expression (11 vs 35 m; p = 0.0609), although the difference was not statistically significant (Fig. 3). Univariate analysis using the cox proportional hazards model was performed for each molecular marker. High expression of CD8 and CD68 was associated with higher PSF (HR = 0.484, 95% CI 0.260–0.903, HR = 0.333, 95% CI 0.147–0.753), OS (HR = 0.376, 95% CI 0.176–0.803, HR = 0.262, 95% CI 0.079–0.868). Multivariate analysis of CD8, CD4, and CD68 expression indicated a significant association with PFS and OS (Table 3). The expression status of other molecules was not associated with the prognosis after ICI treatment (Fig. 4).

**Table 3:** Multivariable analysis association between progression-free survival (PFS)/overall survival (OS) and IHC staining status of CD8, CD4, and CD68.

		Hazard ratio	95% CI		p value
PFS	CD8 +	0.455	0.238	0.868	0.017
	CD4 +	2.009	1.002	4.030	0.049
	CD68 +	0.342	0.149	0.784	0.011
OS	CD8 +	0.267	0.113	0.631	0.003
	CD4 +	3.571	1.486	8.583	0.004
	CD68 +	0.285	0.085	0.954	0.042

**Figure 5:**
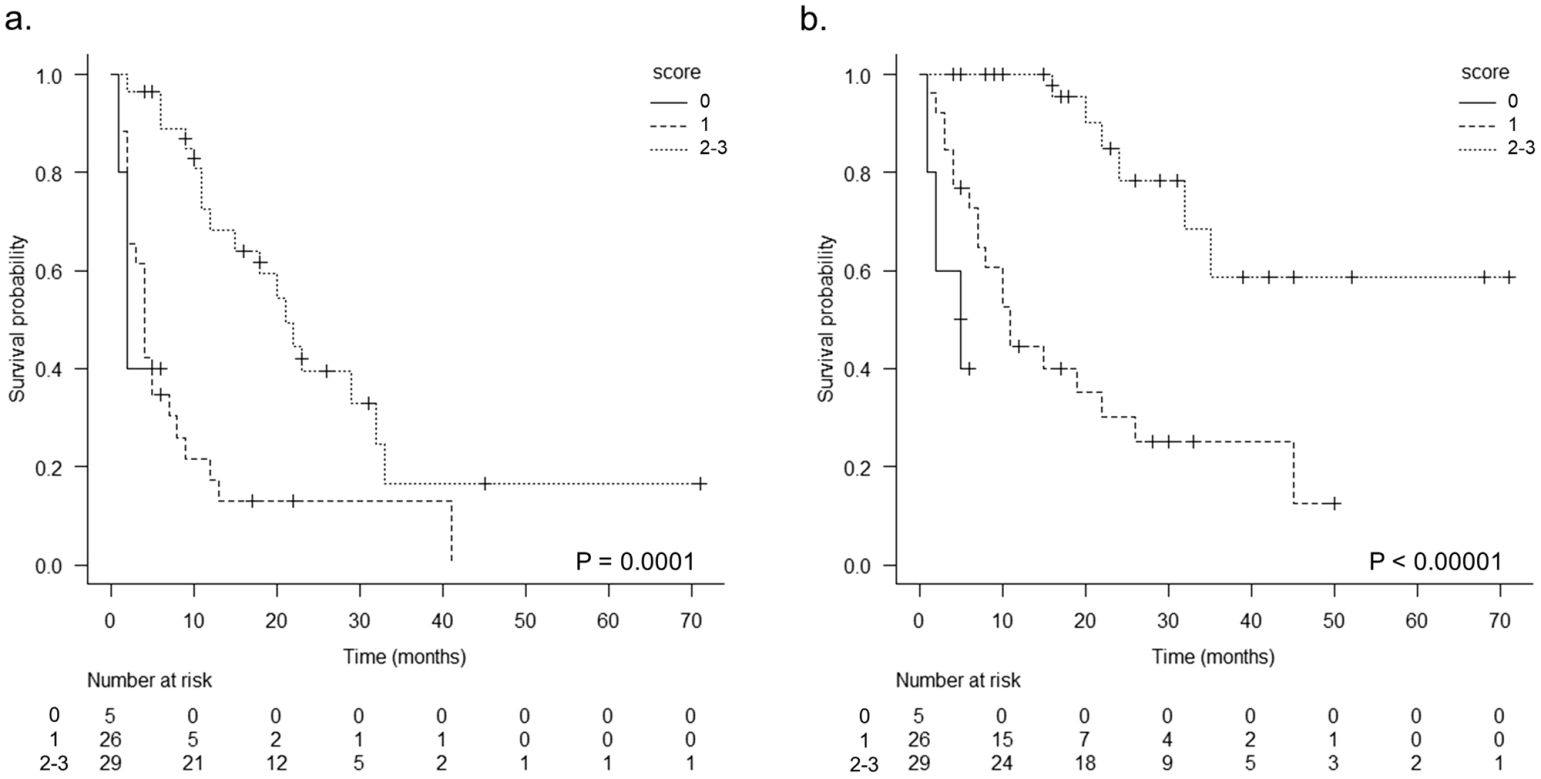
The results of the Kaplan–Meier analysis are presented. Relationship between immune cells score (total score of CD8-positive + 1, CD68-positive + 1 and CD4-negative + 1; Min 0, Max 3) and survival in groups with scores of 0–1 or 2–3 are analysed. (**a**) PFS comparison by immune cells score (log-rank P = 0.0001). (**b**) OS comparison by immune cells score (log-rank P < 0.00001).

**Figure 6:**
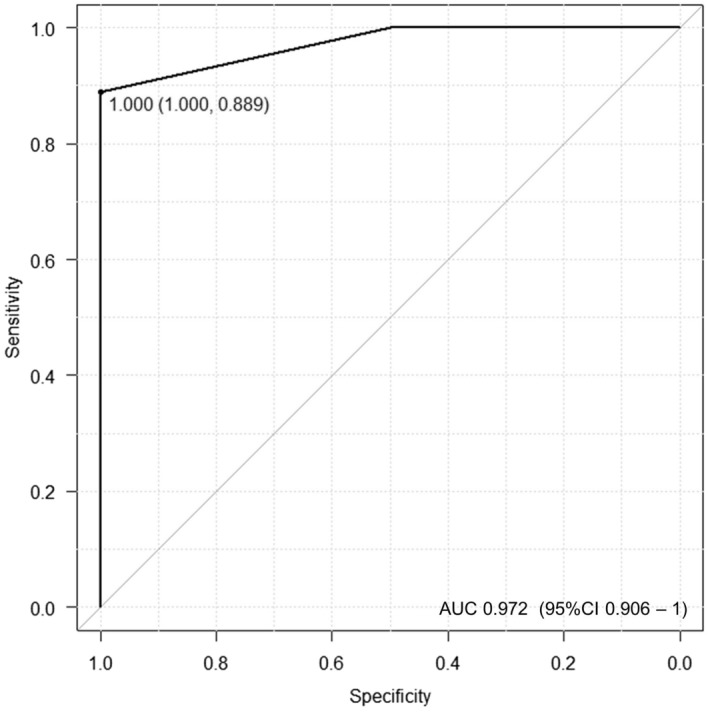
Receiver operating characteristic curve for predicting the response to ICI treatment.

The three markers that were found to be associated with high expression and response to ICI treatment were scored from 0 to 3 as shown below (high CD8 expression; + 1, high CD68 expression; + 1, low CD4 expression; + 1). PFS and OS were assessed in three groups with scores of 0, 1, and 2 or 3 points respectively. The results showed a significantly prolonged prognosis in the group with a score of 2 or 3. (median PFS; 2 m vs 4 m vs 21 m, p = 0.0001, median OS; 5 m vs 11 m vs NR, p < 0.00001) (Fig. 5). Then, we evaluated the validity of the prognostic model using the IHC score. Figure 6 reveals the receiver operating characteristic (ROC) curve derived from the IHC status scoring of CD8, CD68, and CD4 expressions. ROC analysis showed that the area under the curve (AUC) was 0.972 (95% CI 0.906–1.000).

## Discussion

In this study, we investigated several molecules which are associated with tumor immunity using immunohistochemical staining techniques and explored their potential as biomarkers for ICI therapy. We found that high infiltration of CD8-positive T cells in tumor tissue, high expression of CD68-positive tumor-associated macrophages, and low expression of CD4-positive T cells was significantly associated with good response to ICI treatment and survival. These findings may represent a potential biomarker of ICI therapy for patients with RCC, respectively. Furthermore, the combined scoring of the IHC expression status has a powerful potential to be a surrogate marker for a more accurate prediction of ICI treatment efficacy.

CD8-positive T cells constitute a central component of anti-tumor immunity in various malignancies, including RCC^15^. Previous reports have generally suggested that infiltration of CD8-positive T cells is associated with poor prognosis in RCC, such as high grade, high stage, and high recurrence rates^17^. Regarding ICI treatment, high expression of CD8-positive T cells in tumors has been shown to positively associate with favorable clinical outcomes after ICI treatment^18,19^. Exploratory data on CD8 infiltration from the randomized phase III trial JAVELIN RENAL 101 indicated that high CD8 infiltration was associated with poor PFS for patients treated with sunitinib but not for patients treated with the avelumab-axitinib combination^20^. However, there are several opposite reports in RCC^21,22^. This situation demonstrates the peculiarity of the tumor immune environment in RCC. Our results also do not assess in detail the subtypes of infiltrating CD8-positive T cells. Since there are cells termed exhausted T cells^23,24^, which do not have sufficient anti-tumor effects, and in fact, there are subtypes that act pro-tumor^25^. In addition, past studies have shown that CD8-positive T-cells can be divided into three types according to their infiltration pattern into the tumor^26^. Tumors in an uninflamed state, called immune desert or immune exclusion, are particularly resistant to ICI treatment. Therefore, it is important to evaluate not only CD8 expression but also its structural relation with the tumor.

Giraldo et al. highlighted tertiary lymphoid structure(TLS) and dendritic cells in mRCC tissues with high CD8-positive T cells. They reported the importance of their role in effector T-cells activation^27^. We evaluated the correlation between the presence of TLS and ICI treatment by using CD20 as a marker, however, no significant positive association was observed.

CD68 is a protein that is highly expressed in monocytes, blood-circulating macrophages, and tissue-infiltrating macrophages^28^. Tumor-associated macrophages (TAMs) have been reported to be strongly involved in cancer growth in many malignant tumors, and reports from lung cancer^29^, gastric cancer^30^, pancreatic cancer^31^ and breast cancer^32^ have shown that high CD68 expression is positively associated with high tumor grade and T stage and is a poor prognostic factor^33^. Few studies report that a high density of CD68-positive cells is associated with higher tumor grade, higher T stage, and poor prognosis^34^. Based on the mechanism of activation, TAMs can be divided into two types: M1 type, which acts mainly as an anti-tumor growth, and M2 type, which acts mainly as a pro-tumor growth^33,35^. M1 type produces inflammatory cytokines such as IL-6 and IL-12, in contrast, M2 type expresses PD-1 ligand and induces the production of anti-inflammatory cytokines such as IL-10 and TGF-β as well as the proliferation of regulatory T cells^36,37^. Regarding RCC, TAMs are considered to activate cells involved in tumor immunity, such as Tregs, via CXCL20- and IL-10^34,38,39^. Furthermore, high TIM-3 expression in RCC tumors is strongly associated with increased TAMs infiltration, indicating that TIM-3 expression on tumor cells may be regulated by intercellular interactions with TAMs^40^. There are only a few reports on the therapeutic effect of ICI and tumor infiltration of TAM.

In the present study, our data showed that high expression of CD68, a marker of macrophages, was significantly associated with a longer prognosis after ICI treatment. These results deviated from earlier reports of high TAM expression and poor prognosis for RCC. Several recent studies have reported that high TAM expression is a favorable prognostic factor for ICI treatment. Voss et al. reported that M2 macrophage infiltration into tumor tissue was associated with continuous clinical response to anti-PD-1 antibody, by WES and RNAseq analysis^41^. Furthermore, it was very interesting that this trend was not observed in the TKI treatment group. Results of the post hoc analysis of the NIVOREN trial showed that infiltration of CD163-positive macrophages was associated with favorable PFS in patients treated with nivolumab^42^. Therefore, it is possible that tumor-associated macrophages, although a poor prognostic factor in RCC typically, may act beneficially under ICI treatment. It remains unclear how ICI provides an antitumor effect on TAMs, and further investigation is required.

High expression of CD4-positive T cells, especially regulatory T cells (Tregs), has been associated with poor prognosis in some malignancies^43,44^. It has also been reported to be associated with inferior ICI treatment response^45,46^. Our data demonstrated a trend of significantly increased fraction of non-responders in the high CD4-positive T cell expression group. Although there was no association observed between Foxp3 expression, a marker specific for Tregs, and response, it was suggested that the status of CD4 expression could be a biomarker of ICI treatment response in RCC.

There are several limitations in this study. First, it is a relatively small observational study. Compared to the subanalysis data of large clinical trials, the number of cases is limited, and further development into a collaborative study at other institutions with a larger number of cases is expected in the future. In addition, stained images were not analyzed by the objective image analysis software. When strict cutoff values are established with digital data, even immune molecules with previously ambiguous results may become candidates for predicting ICI response. Furthermore, this study included 20 cases of recurrence after nephrectomy, 7 of which were late recurrences that occurred more than 5 years after the initial operation. In these late-recurrence cases, the characteristics of the primary tumor at the time of surgery may differ from those of the metastatic or recurrent lesions. In addition, the status of infiltrating immune cells and the tumor microenvironment may have changed, which is a limitation of this study. To overcome these limitations, a biopsy of metastatic or recurrent tumors is important, taking into account the risks and benefits to the patient. Finally, the heterogeneity of kidney cancers must also be considered. Because the FFPE samples were derived from representative tumor tissue in each patient, the response to ICI treatment and infiltrating immune cells could vary by tumor site in RCC with multiple genotypes.

In summary, we evaluated the relationship between the expression status of various immune-related molecules in RCC and the ICI treatment from multiple perspectives, using the immunohistochemistry technic. We determined that high-expression CD8 and CD68, and low-expression CD4 were associated with more favorable ICI treatment response and prolonged prognosis. Each immune cell and tumor cell balances pro- and anti-tumor via complex crosstalk in the tumor microenvironment. In addition, immune cells regulate their differentiation and proliferation through cytokines and chemokines^47,48^. Therefore, the approach shown here enables a more accurate selection of patients who can benefit from ICI treatment.

## Supplementary Information

Supplementary Information.

## Data Availability

All data generated or analyzed during this study are included in this manuscript.
