# Supplementary material for: Tumor-infiltrating immune cell status predicts successful response to immune checkpoint inhibitors in renal cell carcinoma
Source: Sci Rep. 2022 Nov 27;12:20386. doi: 10.1038/s41598-022-24437-6 (PMC9701769; doi:10.1038/s41598-022-24437-6)
Supplement: Supplementary file 1 — Supplementary Information. [file 41598_2022_24437_MOESM1_ESM.docx]

Supplementary

Primary antibody list

| **Antigen** | **Clone** | **Catalog#** | **Source** | **Dilution** |
| --- | --- | --- | --- | --- |
| CD8 | C8/144B | M731029 | Dako | 1:200 |
| CD4 | 4B12 | M710301 | Dako | 1:100 |
| FoxP3 | 236A/E7 | ab20034 | Abcam | 1:100 |
| CD3 | F7.2.38 | M725429 | Dako | 1:200 |
| PD-L1 | E1L3N | 13684 | CST | 1:200 |
| CD68 | KP1 | M0814 | Dako | 1:200 |
| CD163 | 10D6 | MA511458 | Invitrogen | 1:500 |
| CD57 | NK1 | MA5-12008 | Invitrogen | 1:200 |
| BCL-2 | 124 | MAB11332 | abnova | 1:200 |
| XIAP | 48 | 610762 | BD Biosciences | 1:100 |
| CD20 | L26 | AB9475 | Abcam | 1:200 |

CD; Cluster of Differentiation, FoxP3; Forkhead box P3, PD-L1; Programmed death-ligand 1, BCL2; B-cell/CLL lymphoma 2, XIAP; X Linked Inhibitor of Apoptosis Protein,
